# Total Flavonoids Extracted from Xiaobuxin-Tang on the Hyperactivity of Hypothalamic-Pituitary-Adrenal Axis in Chronically Stressed Rats

**DOI:** 10.1093/ecam/nep218

**Published:** 2011-06-05

**Authors:** Lei An, You-Zhi Zhang, Xin-Min Liu, Neng-Jiang Yu, Hong-Xia Chen, Nan Zhao, Li Yuan, Yun-Feng Li

**Affiliations:** ^1^Beijing Institute of Pharmacology and Toxicology, Beijing 100850, China; ^2^College of pharmacy, Dalian Medical University, Dalian 116044, China; ^3^Institute of Medicinal Plant Development, Chinese Academy of Medical Sciences & Peking Union Medical College, Beijing 100094, China

## Abstract

Our previous studies have demonstrated that the total flavonoids (XBXT-2) isolated from the extract of Xiaobuxin-Tang (XBXT), a traditional Chinese herbal decoction, ameliorated behavioral alterations and hippocampal dysfunctions in chronically stressed rats. Studies over the last decades have suggested that the hyperactivity of hypothalamic-pituitary-adrenal (HPA) axis is one of the most consistent findings in stress-related depression. Herein, we used the same chronic mild stress model of rats as before to further investigate the effect of XBXT-2 on the hyperactivity of HPA axis, including the stress hormones levels and glucocorticoid receptors (GRs) expression. Our ELISA results showed that chronic administration of XBXT-2 (25, 50 mg kg^−1^, p.o., 28 days, the effective doses for behavioral responses) significantly decreased serum corticosterone level and its upstream stress hormone adrenocorticotropic hormone (ACTH) level in chronically stressed rats. Furthermore, western blotting result demonstrated XBXT-2 treatment ameliorated stress-induced decrease of GRs expression in hippocampus, an important target involved in the hyperactivity of HPA axis. These results were similar to that of classic antidepressant imipramine treatment (10 mg kg^−1^, p.o.). In conclusion, the modulation of HPA axis produced by XBXT-2, including the inhibition of stress hormones levels and up-regulation of hippocampal GRs expression, may be an important mechanism underlying its antidepressant-like effect in chronically stressed rats.

## 1. Introduction

Nowadays, depression with increasing morbidity and mortality has become a big health problem to the whole world. Hypothalamic-pituitary-adrenal (HPA) axis, an important neuroendocrine system, has been evidenced as a key structure in the pathophysiology of depression/stress disorder. Studies over the last decades have shown that hyperactivity of HPA axis is the main biochemical change, besides disturbed monoaminergic neurotransmission, observed in patients suffering from major depression [1, 2].

Recently, accumulating studies have showed that herb preparations may provide us a prospective alternative in the treatment of depression for their better compliance and lower side effects, such as *l*-Perillaldehyde (a major component in Perillae Herba) featured by Ito et al. [3], Chaihu-Shugan-San by Kim et al. [4], Banxia-houpu decoction by Li and his colleague [5], and preparations from St John's wort extracts reviewed by Müller [6], but more attention is still needed in this field for deeper and more thorough scientific research.

Xiaobuxin-Tang (XBXT), a traditional Chinese herbal decoction, which contains Haematitum, Flos Inulae, Folium Phyllostachydis Henonis and Semen Sojae Preparatum, four Chinese traditional medicines, was recorded in the silk scroll manuscript of “Fuxinjue Zangfu Yongyao Fayao”, written 1000 years ago. Our previous studies have demonstrated that the total flavonoids (XBXT-2) extracted from Xiaobuxin-Tang (XBXT) exerted potent antidepressant-like effects in multiple animal models of depression [7]. Furthermore, recent studies in our laboratory showed that XBXT-2 (25, 50 mg/kg, p.o., 28 days) could reverse the behavioral alterations and hippocampal dysfunctions in chronically stressed rats, suggesting XBXT-2 may exert significant antidepressant-like effect in chronically stressed rats and the maintenance of hippocampal morphologic and functional plasticity may be involved in it [8, 9]. However, until now, we know nothing about the effect of XBXT-2 on HPA axis in chronic mild stress (CMS) model.

Glucocorticoids (cortisol in humans and corticosterone in rodents), the hormonal end-product of the HPA axis, are hypersecreted in depression/stress disorder, which is one of the most consistent findings in both clinical patients and animal models [1, 2, 10]. Moreover, the upstream stress hormone of glucocorticoids, adrenocorticotropic hormone (ACTH), which is responsible for the release and regulation of glucocorticoids, is also elevated in depressed patients. For example, a significant percentage of depressed patients have increased levels of cortisol in the saliva, serum, urine and cerebrospinal fluid, as well as abnormal 24 h pattern of cortisol and ACTH secretion [11–13]. Similarly, in experimental animals, chronic stress paradigms can recapitulate not only many behavioral characteristics but also lots of biochemical states of depression including elevated corticosterone and ACTH levels [14, 15]. And these alterations can be normalized by most therapeutic agents in clinic, which is in line with their antidepressant response [16, 17].

Besides the elevated stress hormones, the hyperactivity of HPA axis in stress/depression disorder is particularly thought to be related to reduced feedback inhibition by endogenous glucocorticoids. Recently, accumulating studies have indicated glucocorticoid receptors (GRs), one type of glucocorticoids cognate receptors, through which endogenous glucocorticoids serve as potent negative regulators of HPA axis activity, is one of the most important, striking and innovative targets in the pathophysiology and therapy of depression [18–20]. Clinical findings have shown that the number of GRs in CNS was reduced in depressed patients and this might, at least partly, account for the impaired feedback inhibition in HPA axis [21]. A recent research reported transgenic mouse strains that under-express GRs displayed depressive-like behaviors [22]. Furthermore, hippocampus, as an important limbic brain section regulating mood and cognitive functions, has long been considered as an essential regulator in HPA axis negative feedback. Experimental data support the idea that chronic treatment with most successful antidepressant increase the GRs mRNA or GRs protein level in rat hippocampus [23–26].

Considering the crucial role of HPA axis in the pathophysiology and therapy of stress-induced depression, in the present study, we used CMS model, a paradigm causing prolonged HPA axis hyperactivity, to investigate whether chronic XBXT-2 (25, 50 mg kg^−1^, p.o., the effective doses for behavioral responses) administration could decrease the elevated corticosterone and ACTH levels induced by stress, and also, we assessed the effect of XBXT-2 on hippocampal GRs expression in chronically stressed rats.

## 2. Methods

### 2.1. Preparation of the Total Flavonoids from Xiaobuxin-Tang sExtract

#### 2.1.1. Materials

Traditional Chinese medicines Haematitum, Flos Inulae, Folium Phyllostachydis Henonis and Semen Sojae Preparatum were purchased from Beijing Tongrentang Drugstore (Beijing, China) and were identified by Prof. Lian-Sheng Shen, School of Chinese medicine, Beijing University of Chinese Medicine as calcined product of Ochery hematite, flowers of *Inula japonica* Thunb., leafs of *Phyllostachys nigra* (Lodd.) Munro var. henonis (Mitf.) Stapf ex Rendle, fermented product of *Glycine max* (L.) Merr., respectively. The voucher specimens (No. 02002, No. 02003, No. 02004, No. 02005, respectively) are deposited in the Laboratory of Phytochemistry, Beijing Institute of Pharmacology and Toxicology, China.

#### 2.1.2. Extraction

The medicines Haematitum, Flos Inulae, Folium Phyllostachydis Henonis and Semen Sojae Preparatum (2 : 2 : 1 : 1, w/w) (6 kg) were mixed and extracted three times with 70% alcohol at 100°C. The combined extracts were filtered and evaporated by a rotary evaporator under reduced pressure to obtain a viscous alcoholic extract (784 g), which was dispersed in 1 l water and partitioned with petroleum ether. Subsequently, the water fraction was diluted with 4 l water and the water solution was centrifuged at 1560 g for 30 min. Then, the solution was passed by column chromatography on AB-8 macroporous resin (7 l). After eluting with 14 l water, the column was eluted with 21 l 70% alcohol and the 70% alcohol elution was evaporated and dried *in vacuo* to obtain the total flavonoids (320 g). Using lutin as standard, the apparent flavone content of the extract was determined as 76.03% by colorimetric method [27].

### 2.2. Animals

Sprague Dawley rats weighing 180–220 g (Beijing Vital Laboratory Animal Technology Company, Beijing, China) were used for the experimental procedure. The animals were group housed in polypropylene cages under standard experimental conditions: room temperature 21 ± 2°C, humidity 40–60%, 12 h : 12 h light/dark cycle (lights on at 8:00 a.m.). Food and water were available *ad libitum*. Animals were allowed to have a period of acclimation before any experiment. All animal experiments were carried out in accordance with the National Institute of Health Guide for the Care and Use of Laboratory Animals (NIH publication No. 86-23, revised 1996).

### 2.3. Drugs and Reagents

Imipramine hydrochloride (IMI) was purchased from Sigma (St Louis, MO, USA). Rabbit anti-GR (*α* + *β*) and rabbit anti-*β*-actin antibodies were purchased from Santa Cruz Biotechnology (CA, USA). HRP-conjugated goat anti-rabbit IgG antibody was purchased from Zhongshan Technology (Beijing, China). Rat ELISA KIT for corticosterone and ACTH were purchased from TPI Inc (WA, USA).

### 2.4. CMS Procedure

**Figure 1:**
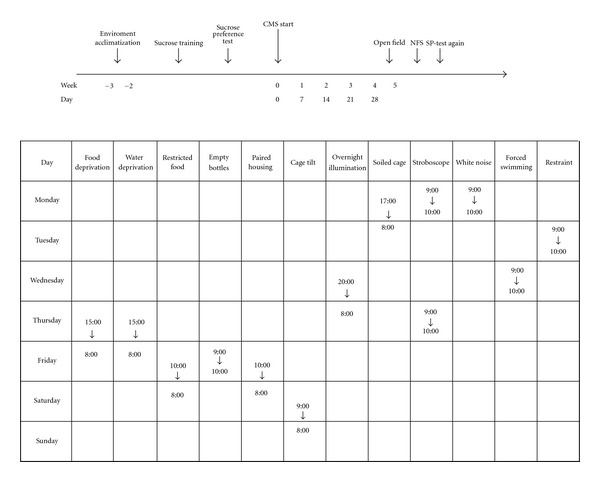
An outline of the design for the experiment (top: with time course for CMS and post-CMS tests; bottom: CMS protocol).

The stress procedure performed here mainly referred to Grønli et al. [28] and Willner [29] with some modifications. The stressed groups were exposed to the following stressors for four consecutive weeks: (i) food or water deprivation; (ii) restricted access to food; (iii) exposure to empty water bottles following a period of water deprivation; (iv) paired housing; (v) cage tilt (45°); (vi) overnight illumination; (vii) soiled cage (200 ml water spilled onto 100 g sawdust bedding); (viii) stroboscopic lighting (100 flashes/min); (ix) white noise (*∼*110 dB); (x) forced swimming (water temperature 15°C, 5 min); (xi) Restraint. The stress procedure in the first week was presented in Figure 1 and repeated with unpredictable sequence during the following 3 weeks. Animals in control group were left undisturbed in the home cages in a separate room. Except “control” group, we divided stressed rats into “stress-vehicle”, “stress-IMI” and “stress-XBXT-2” groups, which received distilled water, IMI (10 mg kg^−1^) and XBXT-2 (25 or 50 mg kg^−1^), respectively. Each group included eight rats. Drugs (including distilled water) were given orally once a day at 8:00–9:00 a.m. from the beginning of stress regime.

### 2.5. Enzyme-Linked ImmunoSorbent Assay

On the 8th day after the end of stress procedure, the rats were decapitated. Blood was sampled, centrifuged (860 g, 20 min) at 4°C and then stored at −20°C. Serum corticosterone and ACTH concentrations were detected by Enzyme Immunoassay (magnetic solid phase) kits (TPI Inc, WA, USA). According to the manufacturer's; protocol, samples (or standard) and conjugate were added to each well, respectively, and then the plate was incubated for 1 h at room temperature without blocking. After several times washing and proper color development, the optical density value was read at 450 nm in ELISA plate reader. The sensitivity of the assay for corticosterone and ACTH was 0.7 nmol/l and 1 pg/ml, respectively.

### 2.6. Western Blotting

Following decapitation, rat hippocampus were isolated rapidly and stored at −80°C for western blotting detection. The tissues were weighed, sonicated in RIPA lysis buffer supplemented with fresh protease and phosphatase inhibitors, and centrifuged at 13 800 g for 25 min. Then the protein concentration was determined by BCA assay. After denaturation and electrophoresis, the proteins were transferred onto a NC membrane in semi-dry conditions. Following blocking in 1% BSA for 2 h, the protein membrane was incubated in primary antibody rabbit anti-GR (1 : 1000) at 4°C overnight and then in secondary antibody HRP-conjugated goat anti-rabbit IgG (1 : 5000) for 1 h. ECL + kit (APPLYGEN Company, Beijing, China) was used for the detection of protein band and the chemiluminescence signal was transformed into a digital image using Kodak films for sequent analysis with software.

### 2.7. Statistics

All data were expressed as mean ± SEM and analyzed by one-way analysis of variance (ANOVA) followed by Newmann-Kuels tests for inter-group comparisons. A value of *P* < .05 was considered statistically significant.

## 3. Results

### 3.1. Reduction of Serum Corticosterone and ACTH Concentration in CMS Rats

**Figure 2:**
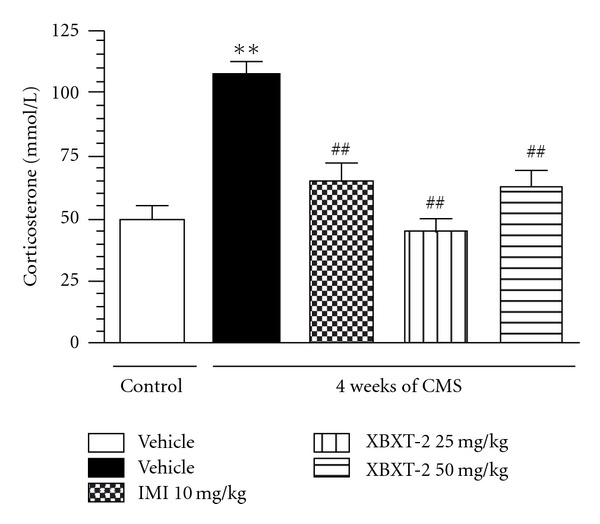
Effect of XBXT-2 on the serum corticosterone level in chronically stressed rats. Serum corticosterone concentration was detected using a commercial ELISA kit. Each column represents as the mean ± SEM, *n* = 8. **P < .01 compared with control-vehicle; ^##^ *P* < .01 compared with stress-vehicle (ANOVA followed by Newmann-Kuels tests).

ELISA analysis demonstrated that 28-day stress exposure significantly increased the serum corticosterone concentration in rats by 110.38% (*P* < .01, versus control-vehicle). Chronic XBXT-2 (25, 50 mg kg^−1^, p.o.) or IMI (10 mg kg^−1^, p.o.) treatment, as shown in Figure 2, attenuated this alteration and decreased serum corticosterone concentration by 56.89%, 40.47% and 40.04%, respectively (*F*(4,35) = 16.77, *P* < .01 versus stress-vehicle).

**Figure 3:**
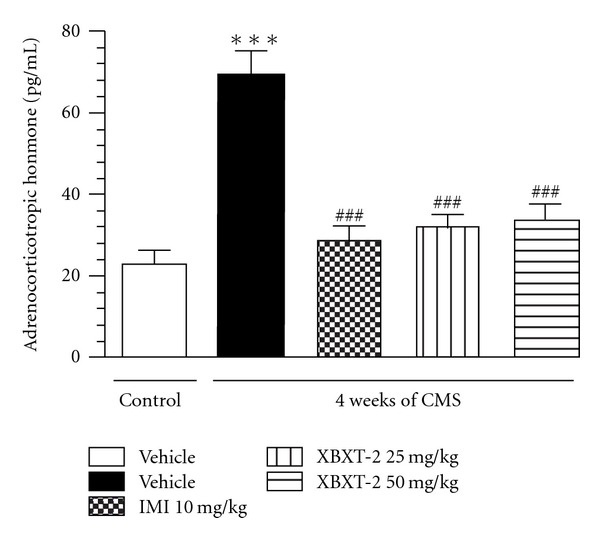
Effect of XBXT-2 on the serum ACTH level in chronically stressed rats. Serum ACTH concentration was detected using a commercial ELISA kit. Each column represents as the mean ± SEM, *n* = 8. ***P < .001 compared with control-vehicle; ^###^ *P* < .001 compared with stress-vehicle (ANOVA followed by Newmann-Kuels tests).

As shown in Figure 3, serum ACTH concentration was significantly increased in chronically stressed rats by 198.03% compared with control (*P* < .001). In line with its effect on corticosterone concentration, chronic XBXT-2 (25, 50 mg kg^−1^, p.o.) administration reversed this change and reduced the ACTH concentration by 53.38% and 51.01%, respectively (*P* < .001 versus stress-vehicle). Similar result was observed in IMI group (*P* < .001 versus stress-vehicle).

These two results indicated that chronic XBXT-2 treatment significantly decreased the stress hormones (corticosterone and ACTH) levels, which were remarkably elevated by the stress regime.

### 3.2. Up-Regulation of Hippocampal GRs Expression in CMS Rats

**Figure 4:**
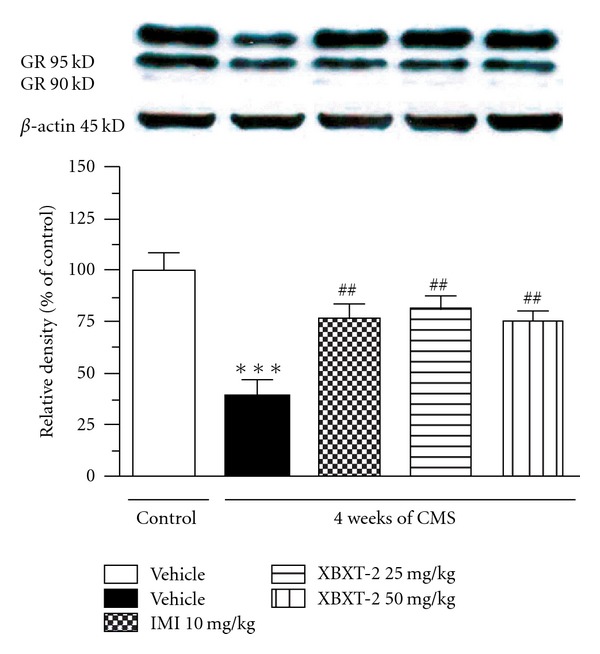
Effect of XBXT-2 on hippocampal GRs expression in chronically stressed rats. The intensity of western bands was quantified with a densitometric scanner. Each column represents as the mean ± SEM, *n* = 5. Band 1: control + vehicle; Band 2: stress + vehicle; Band 3: stress + IMI (10 mg/kg, p.o.); Band 4: stress + XBXT-2 (25 mg/kg, p.o.); Band 5: stress + XBXT-2 (50 mg/kg, p.o.); ***P < .001 compared with control-vehicle, ^##^ *P* < .01 compared with stress-vehicle (ANOVA followed by Newmann-Kuels tests).

Western blotting analysis (Figure 4) showed that the total GRs expression in hippocampus were decreased by 28 day stress regime (*P* < .001 versus control-vehicle). While chronic XBXT-2 (25, 50 mg kg^−1^, p.o.) or IMI (10 mg kg^−1^, p.o.) administration significantly increased hippocampal GRs expression [*F*(4,20) = 9.618, *P* < .01 versus stress-vehicle].

## 4. Discussion

Our previous work have shown that the 28-day unpredictable stress paradigm induced a series of depressive-like behaviors in rats, including reduced sucrose preference, deficient locomotor activity, prolonged latency to novelty-suppressed feeding and weight loss, which were consistent with many previous reports [8, 15, 29, 30], and chronic XBXT-2 (25, 50 mg kg^−1^, p.o., 28 days) treatment significantly reversed these alterations, and also in present study we got similar behavioral results as before (not listed). Furthermore, we found that the stress hormones including corticosterone and ACTH levels in rats were significantly increased by chronic stress exposure, as well as the decreased hippocampal GRs expression, which together manifested the hyperactivity and impaired feedback inhibition of HPA axis in chronically stressed rats. Similar to the behavioral effects, chronic XBXT-2 at dose of 25 or 50 mg kg^−1^ (p.o.) treatment significantly attenuated serum hormones levels and increased GRs expression in hippocampus. Based on these data, the authors conclude that the effect of XBXT-2 exerted on the stress-induced HPA axis hyperactivity may be an important mechanism underlying its antidepressant-like effects.

As evidenced by enormous studies, hyperactivity of HPA axis, which is commonly seen in depressed patients, could be reversed during clinically effective therapies with antidepressant drugs [2, 17]. Moreover, among a multitude of molecular events, the stress hormones levels and hippocampal GRs number are two important targets by which antidepressant drugs may exert their clinical effects [16, 31].

Stress—a well-known environmental factor capable of precipitating or exacerbating depressive episodes in humans—is also a strong trigger of HPA axis activation [32]. The HPA axis, as the name implies, consists of a feedback loop including the hypothalamus, pituitary and adrenal glands. Briefly, the hypothalamus releases corticotropin-releasing hormone and arginine vasopressin in response to a stressor, which in turn activate the secretion of ACTH from the pituitary, which finally stimulates the secretion of cortisol (in humans) or corticosterone (in rodents) from the adrenal cortex. As observed in our experiment, 28-day stress significantly increased serum corticosterone and ACTH levels in rats, accompanied by the depressive-like behavioral alterations (not list, similar as [8]), while XBXT-2 (25, 50 mg  kg^−1^, p.o., 28 days) administration significantly decreased the elevated serum corticosterone and ACTH levels, which may account for a primary neuroendocrine mechanism underlying its behavioral effects.

Hippocampus, known to contain high density of GRs, is the key structure in the inhibition of stressed-induced HPA axis activation, and also more sensitive to glucocorticoid-mediated impairment [31, 33]. Indeed, it has been suggested that prolonged overproduction of glucocorticoids, whether as a result of ongoing stress or a genetic predisposition to HPA axis hyperactivity, damages GRs, especially in the hippocampus which is essential for HPA axis restraint, and in turn reduces GR-mediated negative feedback by endogenous glucocorticoids, which, on the other hand, promotes the sustaining hypersecretion of glucocorticoids (this process is also known as the glucocorticoid cascade hypothesis) [2]. In animals, chronic stress paradigms have been evidenced to down-regulate hippocampal GRs number [34, 35]. Likely, in the present study, we found the hippocampal GRs expression in rats was significantly reduced by chronic stress procedure, in another word, impaired by the oversecretion of corticosterone, and chronic XBXT-2 treatment reversed this change, which may account for a more profound neuroendocrine mechanism underlying its behavioral effects.

**Figure 5:**
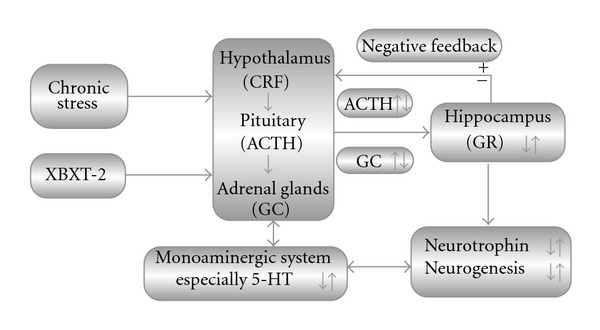
Main molecular targets of XBXT-2 that underlie its antidepressant-like effect. Arrows indicate down-regulation or up-regulation. CRF, corticotropin-releasing factor; ACTH, adrenocorticotropic hormone; GC, glucocorticoids; GR, glucocorticoid receptors.

In mammals, the HPA axis and the 5-HT system are closely interacted in CNS (particularly in hippocampus) and greatly involved in stress-related disorders [36, 37]. Given that 5-HT in hippocampus is highly sensitive to glucocorticoids, it is not surprising that chronic hypercortisolism could cause dysfunction of 5-HT nervous system especially in hippocampus [38]. In our previous work, we have demonstrated that chronic XBXT-2 administration significantly increased hippocampal 5-HT and its metabolite 5-hydroxyindoleacetic acid levels in chronically stressed rats [8]. Therefore, it could be speculated that the amelioration XBXT-2 produced on the monoaminergic system may be associated with its effect on HPA axis. Meanwhile, extensive literature have shown that hyperactivity of HPA axis or sustaining hypersecretion of glucocorticoids may result in hippocampal atrophy in major depression, including dendritic shrinkage, impaired neurogenesis and deficient neurotrophic factors [39–41]. Studies in our laboratory have shown that chronic XBXT-2 treatment increased hippocampal neurogenesis, and also hippocampal BDNF and pCREB (Ser133) expression in chronically stressed rats [9]. Accordingly, it seems that the beneficial effects that XBXT-2 produced on the hippocampus may, at least partly, arise from its regulation on the hyperactivity of HPA axis. Taken together, the aforementioned mechanisms produced by XBXT-2 are not contradictory, but supported and connected with each other (Figure 5). Briefly, in the authors' point of view, XBXT-2 played a significant role in the modulation of HPA axis function, which may be an important and essential mechanism underlying its antidepressant-like effects in chronic stress model of rats.
